# Supplementary material for: Chromothripsis during telomere crisis is independent of NHEJ, and consistent with a replicative origin
Source: Genome Res. 2019 May;29(5):737–49. doi: 10.1101/gr.240705.118 (PMC6499312; doi:10.1101/gr.240705.118)
Supplement: Supplemental Material [file supp_gr.240705.118_Supplemental_file_1.zip › contigs/annotated_contigs/DB94/contig.2.DB94_length_269_mean_cov_4.0780669145.docx]

**DB94_length_269_mean_cov_4.0780669145**

```
TGCCTGGCTAATTTTTGTATTTTTGGTAGAGGTGGGTTTCACCATATTAGCCAGGCTGGTCTAGAATGCCTGGCCTTAAGTGATCCGCT
 >chr8:21109719-21109852 - E=1e-44
CCCCTCGGCTTCCCAAAGTGCTGGGATTACAGGCGTAAGCCACC|GCGCCCGGCCAAAG|TTTTTTTTTATTTATAAAATAGGGATAAT
                                                           >chr9:102849181-102849303 -
GTAATTCCTACTTCACGTTCAGAGTTGCTATGATGATTTAAAGAGATAATATACATGTTTGTAAACTGTTGATAGAAGATACTGTTAGA
E=3e-62
TTAA
```
